# PC4C_CAPSI: Image data of capsicum plant growth in protected horticulture

**DOI:** 10.1016/j.dib.2024.110735

**Published:** 2024-07-10

**Authors:** Namal Jayasuriya, Oula Ghannoum, Wen Hu, Norbert Klause, Weiguang Liang, Yi Guo

**Affiliations:** a Hawkesbury Institute for the Environment, Hawkesbury Campus, Western Sydney University, Richmond, NSW 2753, Australia; b National Vegetable Protected Cropping Centre, Hawkesbury Campus, Western Sydney University, Richmond, NSW 2753, Australia; c Centre for Research in Mathematics and Data Science, School of Computer, Data and Mathematical Sciences, Western Sydney University, Parramatta, NSW 2150, Australia; d School of Computer Science and Engineering, The University of New South Wales, Sydney, NSW 2052, Australia

**Keywords:** RGBD, Capsicum Annum, Glasshouse, Single camera, Time series, Growth cycle

## Abstract

Feeding the increasing global population and reducing the carbon footprint of agricultural activities are two critical challenges of our century. Growing crops under protected horticulture and precise crop monitoring have emerged to address these challenges. Crop monitoring in commercial protected facilities remains mostly manual and labour intensive. Using computer vision to solve specific problems in image-based crop monitoring in these compact and complex growth environments is currently hindered by the scarcity of available data. We collected an RGBD dataset for vertically supported, hydroponically-grown capsicum plants in a commercial-scale glasshouse facility to fill this gap. Data were collected weekly using a single top-angled stereo camera mounted on a mobile platform running between the hydroponic gutters. The RGBD streams covered 80 % of the crop growing season in three different light conditions. The metadata include camera configurations and light condition information. Manually measured plant heights of ten selected plants per gutter are provided as ground truth. The images covered the whole plants and focused on the top third. This dataset will support research on plant height estimation, plant organ identification, object segmentation, organ measurements, 3D reconstruction, 3D data processing, and depth noise reduction. The usability of the dataset has been successfully demonstrated in a previously published study on plant height estimation using machine learning and 3D point cloud.

Specifications table

**Table:** 

Subject	Horticulture, Computer Vision and Pattern Recognition, Applied Machine Learning
Specific subject area	Computer vision, pattern recognition and 3D point cloud processing for structural measures and organ identification of vertically supported capsicum crop.
Data format	Raw data
Type of data	RGBD stream (.bag), Metadata (.json)
Data collection	The Intel Realsense D415 depth camera module with a vertical top angled view and Realsense Viewer software installed on a laptop were mounted on a mobile platform which moved manually on the rail system of the state-of-the-art glasshouse facility. Data were collected weekly over 20 weeks for one side of the hydroponic gutters in three glasshouse rooms with different light filters (scattered light, Smart Glass film, LLEAF film). For each data collection, the angle of the camera was measured and the height of ten plants was manually measured. Manually measured and extracted data were stored as json files, and RGBD streams and meta data were stored in Rosbag files as supported by the Realsense viewer software.
Data source location	National Vegetable Protected Cropping Centre (NVPCC), Hawkesbury Campus, Western Sydney University, Richmond, NSW 2753, Australia
Data accessibility	Repository name: Image Data of Capsicum Plant Growth in Protected Horticulture: PC4C_CAPSI. [1] Data identification number: 10.26183/1A0R-E318 Direct URL to data: https://rds.westernsydney.edu.au/Institutes/HIE/2024/Jayasuriya_N/ Instructions for accessing these data: Access the data repository using the provided URL and the page will show the data description and data storage file system. Data are compressed at the glasshouse room and month level to make it easier to download. The size of each zip file varies from 12GB to 25GB. Realsense Viewer software can be used to replay the recorded streams in 2D or 3D format. The software allows visualising frame metadata and saving a frame as an image. For initiating data processing, a GitHub repository is provided that allows data loading, image extraction, depth post-processing, depth error correction, 3D reconstruction, and visualisations [2]. GitHub link: https://doi.org/10.5281/zenodo.12044907
Related research article	Machine vision based plant height estimation for protected crop facilities, by N. Jayasuriya et al. [3] Link to Paper: https://doi.org/10.1016/j.compag.2024.108669

## 1 Value of the Data

• Images from vertically support crops grown in state-of-the-art commercial-scale high-tech glasshouses are still not common; these facilities have access restrictions, and data collection for the full growth cycle is time consuming, and requires crop specific hardware. This article describes such a dataset for computer science researchers to contribute to automated crop monitoring.
• Plant height has been identified as a proxy measure of plant growth. This dataset can be used to improve the method proposed in our original research paper on plant height estimation or to propose new methods.
• Plant organ identification, size estimation, and total leaf area estimation are some other measures of plant growth. This dataset can be used for organ identification and segmentation with computer vision and machine learning techniques. Size estimations can be done using the 3D scenes built from depth images.
• Object tracking is another area of computer vision and pattern recognition research and is primarily focused on tracking of humans, animals, and vehicles. This complex crop environment dataset can be used to develop robust object tracking algorithms for plant tracking.
• Low-cost depth cameras generally have depth noise problems. This RGBD dataset provides a wealth of depth information of complex scenes along with RGB images. Computer vision and machine learning techniques can be used for depth noise reduction, hole filling of depth images, and for improving / processing 3D point clouds.

## 2 Background

Farming is moving toward precision and protected agriculture, targeting increased productivity and quality produce using less resources and mitigating impacts of climate change [4]. Protected horticulture involves complex and compact crop architectures and maintenance practices that require the participation of skilled labour [5]. Image-based monitoring of plants is largely carried out for research purposes for small and movable plants, while there are limited data using the less accessible crops in commercial facilities [6]. Vertically supported crops in commercial-scale glasshouses are rarely the focus of image-based monitoring due to the complexity and limited accessibility of such environments for researchers [7]. RGB images are the most common, cost-effective, and useful image type for crop monitoring, providing greater usability with associated data for 3D reconstruction [8]. This article presents an RGBD dataset of a vertically supported capsicum crop grown in a commercial-scale glasshouse. Our recent work [3] presents a case study in using this dataset to accurately estimate plant height employing machine learning and 3D information processing. This data article complements our published work and helps improve future research targeting commercial-scale crop monitoring as well as computer vision research.

## 3 Data Description

**Fig. 1:**
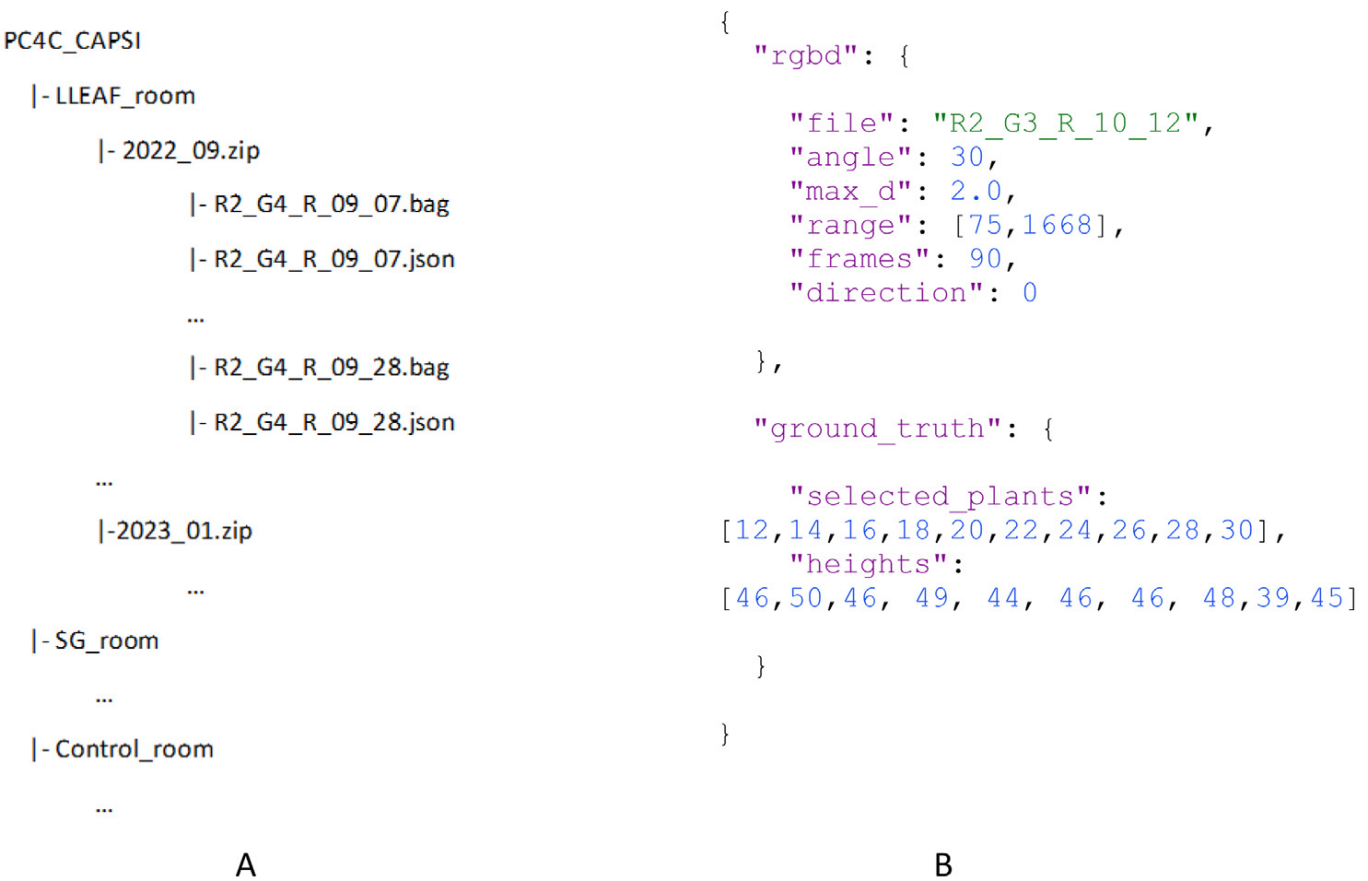
A: Directory structure of data set, B: Content format of .json files.

The dataset PC4C_CAPSI consists of RGBD streams and their meta data of a capsicum crop grown in three glasshouse rooms under three different light filters. Data were collected from the right side of one plant row in a room, and for 20 weeks on average. The organization of the data in the storage is shown in Fig. 1A. The first level of the folder structure defines the glasshouse compartment which differs in their light condition, the next level is month, and on average four time points of data per month are there. For a single data point there are two file types, a Rosbag (.bag) file and a Json (.json) file. In total, there are 59 Rosbag and Json file pairs (17 in the room with LLEAF film and 21 for each of the other two rooms), targeting 105 plants (35 plants in each room and for three rooms). As the data collection platform was manually moved, the length of a stream has varied and on average it was 44 s between 2nd to 36th plant which were targeted plants.

A Rosbag file contains RGBD streams which is 15 frame per second, RGB frame meta data, depth fame metadata, and the camera metadata for 3D reconstruction. The Rosbag file format was used as it is the default format of storing stream data using the Realsense software and the software allow to replay the Rosbag files. This replaying imitates the real time visualization of data while they were collecting. The metadata of the RGB image includes timestamp, image size, frame rate, frame number, exposure settings, light condition settings, and adjustments to the image, while metadata of depth image have the same metadata excluding adjustments to the image and includes laser emitter settings. The timestamp can be converted to date and time up to seconds with its first ten digits using any timestamp converter. A detailed list of these metadata is listed in Table 1 with example values. In addition to the RGB and depth image metadata, camera intrinsic and depth scale can be extracted for reconstruction of 3D scene, and their extraction is explained in the methodology.

**Table 1:** RGB and depth image metadata.

RGB image metadata		Depth image metadata	
Parameter	Sample value	Parameter	value
Frame Timestamp	1,665,556,269,343.5	Frame Timestamp	1,665,556,269,342.9
Clock Domain	Global Time	Clock Domain	Global Time
Frame Number	46,948	Frame Number	46,946
Hardware Size	1280×720	Hardware Size	1280×720
Display Size	1280×720	Display Size	640×360
Pixel Format	RGB8	Pixel Format	Z16
Hardware FPS	14.89	Hardware FPS	14.89
Viewer FPS	15.85	Viewer FPS	14.39
Frame Counter	46,948	Frame Counter	46,946
Frame Timestamp	429,302,422	Frame Timestamp	429,301,821
Sensor Timestamp	427,747,139	Sensor Timestamp	429,293,409
Auto Exposure	1	Actual Exposure	1704
Time of Arrival	1,665,556,269,470	Gain Level	16
Backend Timestamp	1,665,556,269,399	Auto Exposure	1
Actual Fps	14	Actual Fps	15
Brightness	0	Time of Arrival	1,665,556,269,420
Contrast	50	Backend Timestamp	1,665,556,269,367
Saturation	64	Frame Laser Power	300
Sharpness	50	Frame Laser Power Mode	1
Auto White Balance Temperature	1	Exposure priority	1
Backlight Compensation	0	Exposure ROI Left	160

Manually measured camera angle, travel direction of the imaging platform, and a few processing parameters such as starting and ending frame IDs for cropping the stream, number of frames to extract, and maximum depth are stored under the “rgbd” key in the .json file with the same name as the .bag file. Under the “ground_truth” key, selected plant IDs and their heights are stored. An example of content in a .json file is shown in Fig. 1B, and the definitions of the keys are defined below. The name of the corresponding Rosbag file is stored under key of “file”, angle between camera pole (vertical axis) and camera plane is stored under key of “angle”, maximum cap of depth value for further processing is defined as “max_d”, the key “range” defines the selected range of fames for targeted plants, “frames” is the key for number of RGB and depth frame pairs that is target to extract from the Rosbag file and the key “direction” denotes moving direction of platform while collecting data. The values of keys: “max_d”, “range”, and “frames” are defined by replaying the corresponding .bag file and manually investigating it targeting the research requirements of further processing. Hence, these values are subject to change by the researcher who uses the dataset.

*file : file name of the corresponding .bag file*
*angle: angle between the vertical axis and the camera plane*
max*_d: manually investigated maximum depth to cover foreground plant line.*
*range: manually selected starting and ending frame IDs to crop stream.*
*n_frames: the number of frames needed to extract for further processing.*
*direction: the moving direction of the imaging platform (0: plant 1 – 40, 1: plant 40 - 1)*
*selected plants: selected plants for manual height measurements*
*heights: height measurements of selected plants (each height belongs to plant ID in the same index of selected plants)*

**Fig. 2:**
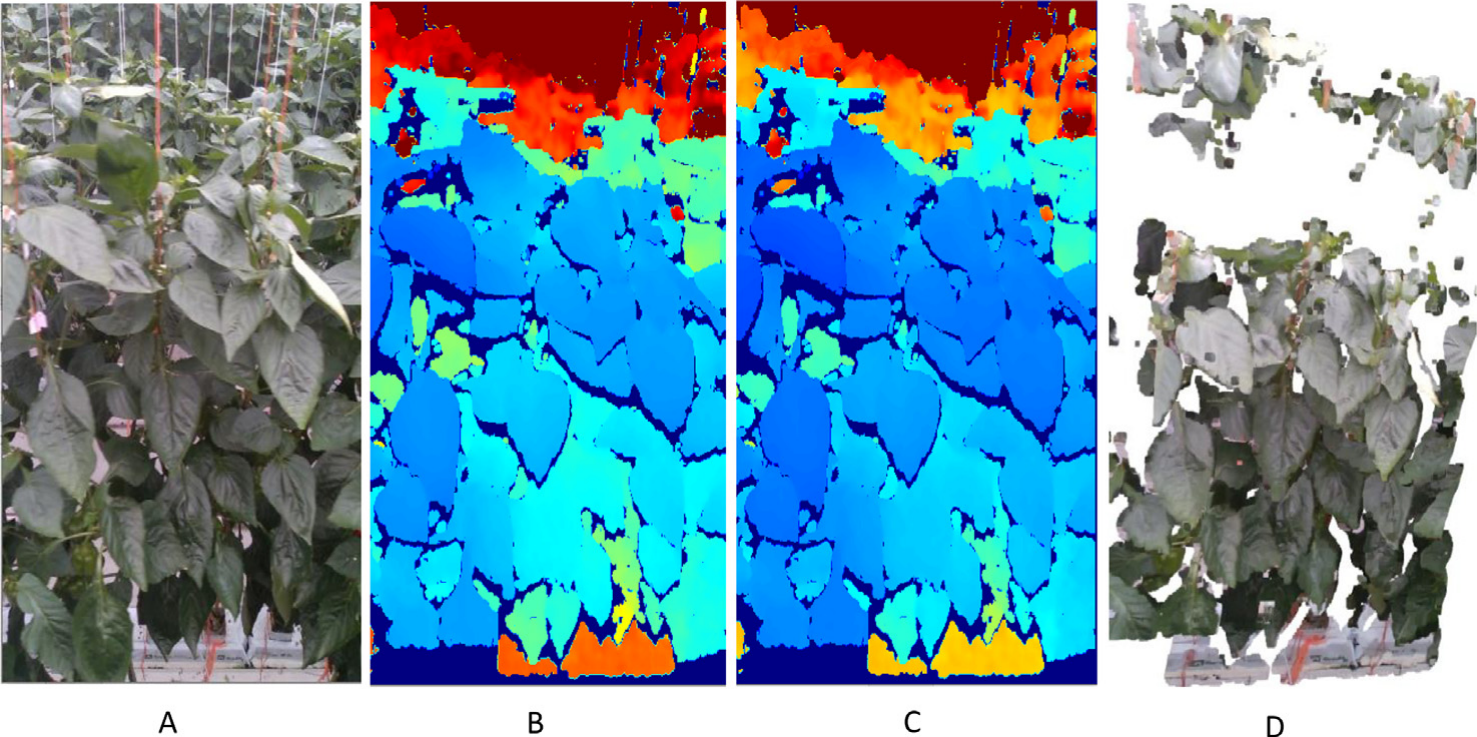
A. Sample RGB image, B. Colorised visualisation of depth image, C. Corrected depth image, and D. 3D reconstructed colored scene with corrected depth.

The RGBD data streams provide RGB images and depth images of a scene. The RGB image (Fig. 2A) can be used to visually recognize the plants, organs, or any other visual features of plants, while each value of the depth image pixel provides the distance from the camera plane to the object seen in that pixel. Depth information can be used directly or to 3D reconstruct the scene and can also be integrated with RGB images to generate a colored 3D scene (Fig. 2D). A calibration fault of the camera was observed later, and a dataset from a calibration correction experiment was used to correct the depth values in depth images. An example depth image and a corrected version of same depth images are shown in (Fig. 2B) and (Fig. 2C), respectively, while the calibration correction methodology is explained in the methodology section. The color ranges from cool (blue) to hot (red) shows the near to distant depth values, respectively, and increased depth error with the distance can be visually observed by comparing original and corrected depth images.

**Fig. 3:**
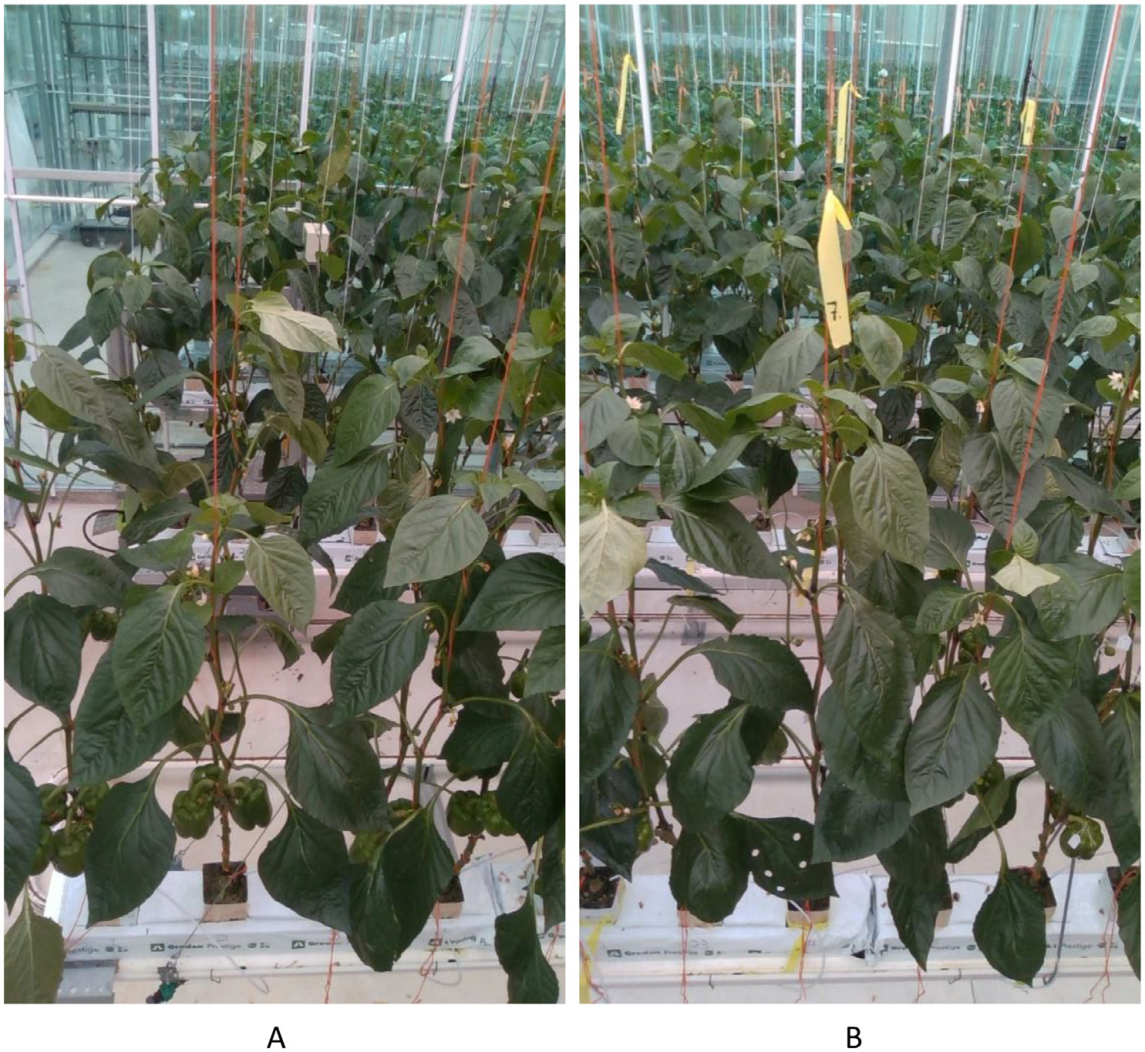
A. Identification of start of plant gutter and B. identification of selected plants.

**Fig. 4:**
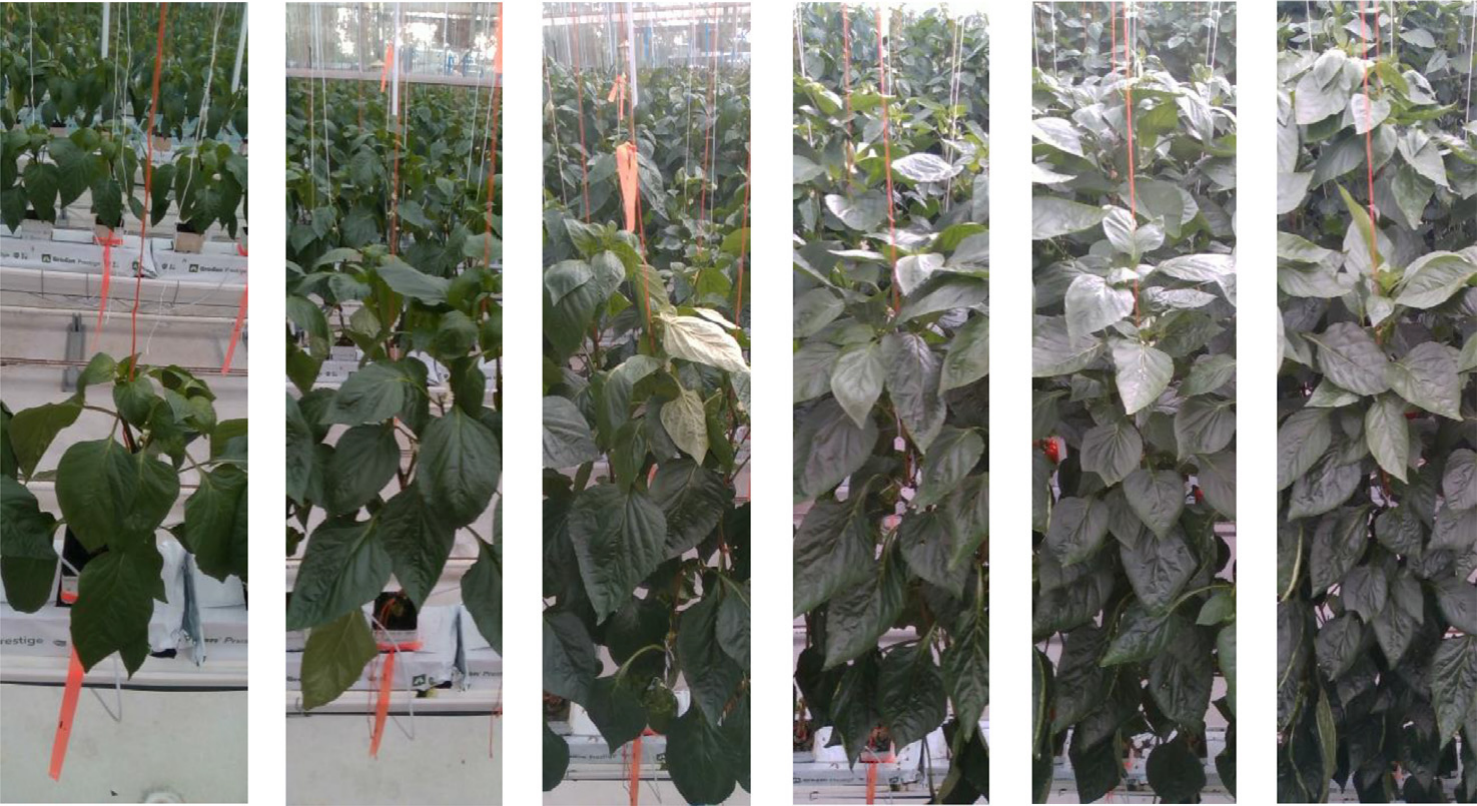
Visualization of growth of a plant over time for each 4 weeks.

Determining plant identification numbers and selected plants that were used for manual height measurements are important in data processing. The plants have been numbered in chronological order (can be seen on the rockwool base that plants are growing in and just below the rockwool cube); however, they can be hardly visible in images taken with top angled view when plants have grown very tall. Hence, an alternative technique was needed. Each plant was vertically supported by two main branches of the stem (orange color strings in Fig. 3). The beginning of the plant gutter is also visible in the images at the beginning of a stream (Fig. 3A). The plant top or rockwool cube at the base (if visible) can be used to identify a plant while replaying the recorded RGBD streams and counting from the beginning of the gutter will help to identify the plant ID of each plant. Ten plants were selected with even plant IDs from 12th to 30th plant and they were tagged with a color strip with a number at the bottom and top of the plants (yellow strips in Fig. 3B, and orange strips in Fig. 4). Here, although the plant Ids are even numbers from 12 to 30 considering the plant line, the color strips are labeled considering only the selected plants 1–10. Hence, 12th plant is the first selected plant, 14th plant is second selected plant and so on. These labelled strips can be used to identify selected plants and in case the strip is difficult to recognize, the above-mentioned counting method can be used from the beginning of the gutter or from the nearest identified plant.

The crop cycle consists of six months after transplanting the seedlings and data collection started two weeks after transplantation. The average crop growth was around 10 cm per week, The canopy became dense and difficult to distinguish neighboring plants after 12 weeks of growth. Top one-third of the canopy is visible in this dataset throughout the crop cycle including some fully visible leaves in that area. The lower canopy and base area become less visible with the growth. Fig. 4 visualizes the growth cycle, density and visibility of lower canopy of the 12th plant over time, starting 2 weeks after transplantation and then with 4-week intervals. Fruits are often not visible because of occlusion and being mostly in the middle and lover canopy. Although Fig. 4 shows the same plant over time, it is quite difficult to identify it as the same plant. The reasons include plant growth, pruning, damages to leaves due to moving of scissor lifts, sample collection, and winding up of the plant by the supporting wire each week. After winding up the growing plants and following pruning, leaves from same and nearby plants, which were previously hidden, became visible and appeared differently. Hence, there were only two options for identifying a selected plant: using the color strips and counting from the beginning of the plant line while replaying the stream.

Another complex part of data is identifying plant tops. The appearance of plant tops can be diverse due to several reasons such as, stresses, occlusion, and activities such as pruning and winding up using supporting wires. Table 2 describes most of the diversities of plant tops, helping researchers to identify them. The observed different types of plant tops are clearly visible tops with a few noticeably young leaves, complexity with side view, partially visible top due to a relatively large leaf, appearance of branching at the top of the stem, abnormality due to winding up the plants, stressed plant tops, and complex and blurred captures. Each row of the table shows the variations under each criterion.

**Table 2:** Complexity of plant top identification.

Observed Type	Examples

## 4 Experimental Design, Materials and Methods

### 4.1 Specification of infrastructure, crop, and camera setup

**Fig. 5:**
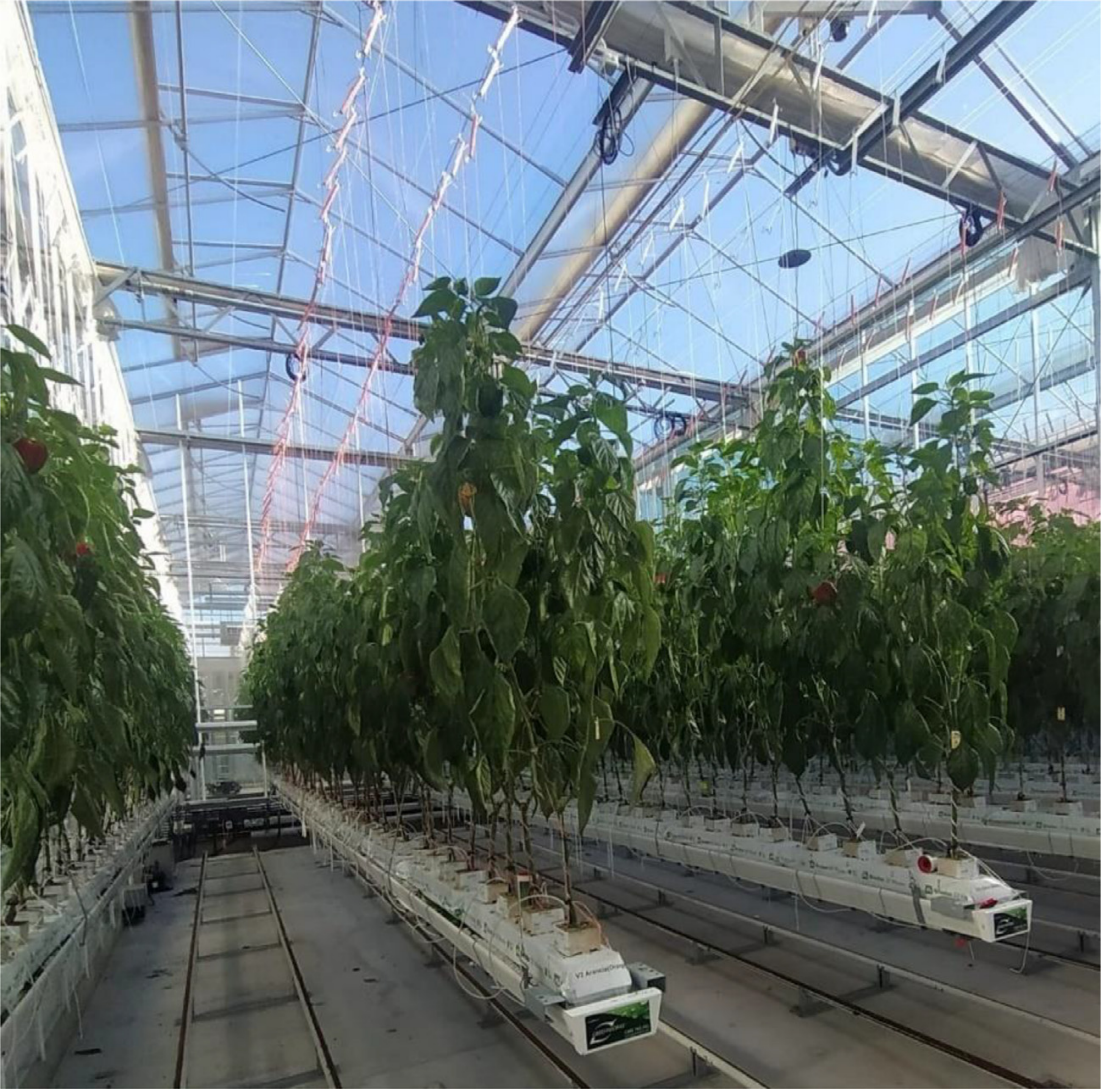
Glasshouse compartment. Reused from Fig. 2 of [3], with permission.

The data collection was carried out at the National Vegetable Protected Cropping Centre (NVPCC) at the Hawkesbury campus of Western Sydney University. The NVPCC is a state-of-the-art smart glasshouse facility consisting of a large glasshouse compartment for teaching purposes and eight research purpose compartments. Each compartment (enclosed area of 105 m^2^) consisted of light diffusing glass roofs that diffuse 70 % of light and walls that diffuse 5 % of light. Additionally, the LLEAF film [9] was installed in two rooms and the Smart Glass film was installed in two other rooms and rest is without any additional films. For this data collection only three rooms were used, a room with LLEAF film, a room with Smart Glass film and a room without any additional films. The LLEAF film shifts the green light spectrum which is less usable for plants towards the red spectrum, and this also increases the amount of near infrared (NIR) as the shifting margins are not sharp. The Smart glass films that aim to reduce heat transfer into indoor environment, block 86 % of ultraviolet, 26 % of red, and 58 % of the far red spectrum [10]. Each room was equipped with an electronically foldable roof curtain to protect plants from high intense radiation. The facility was controlled by a computerized climate control system that controls temperature, humidity, CO_2_ concentration; fertigation management system which provides control over the volume of water and nutrition mixture. Rockwool was used as the growth media in the facility, and plants are organized as rows on the gutters that hang from the ceiling. There were six gutters in each room and each gutter can accommodate 10 rockwool slabs (100 × 15 × 10 cm, Gordan, the Netherlands) and seedlings grown on rockwool cubes (10 cm x 10 cm x 7 cm) were transplanted onto slabs where one slab accommodates 4 plants. On the floor, between each two gutters there was a pipe rail system for the flow of heated water to increase temperature in winter and to move trollies and scissor lifts to manage the crop. The components of a glasshouse compartment can be visually seen in Fig. 5. The seedlings used for this data collection were *Capsicum annum* L. (red Gina variety) donated by Syngenta Australia [11]. The plants were grown up to 2.5 m and they were supported by nylon wires attached to the ceiling. Each plant was managed to grow with two main stems by pruning additional branches, dead leaves, and extra leaves to control the crop density.

**Fig. 6:**
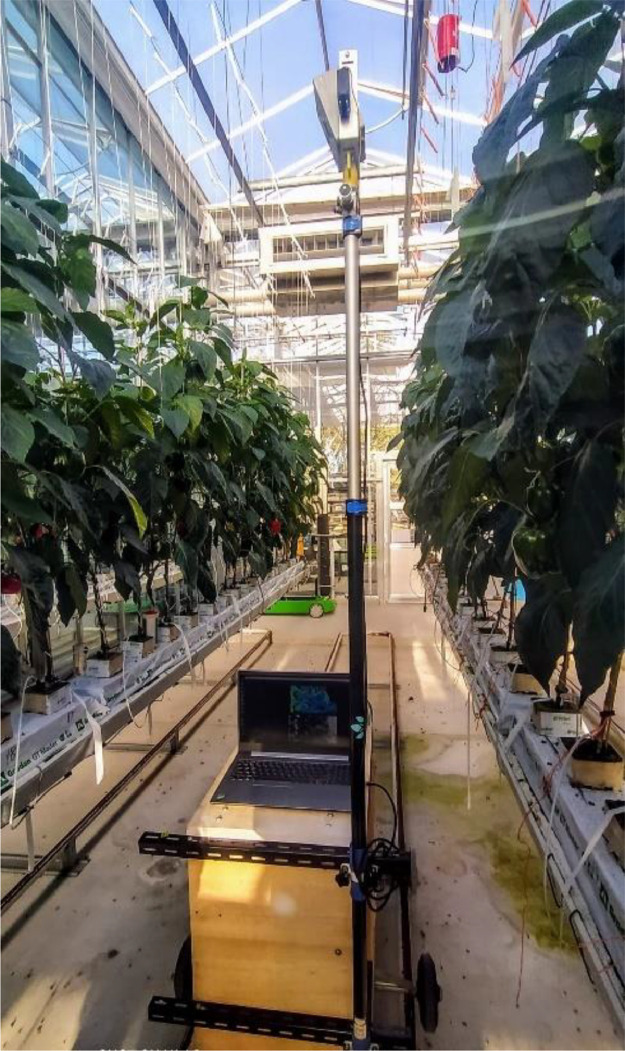
Imaging platform while collecting data in glasshouse. Reused from Fig. 3(a) of [3], with permission.

The imaging platform was placed on top of a greenhouse trolley, which allowed us to adjust camera height and its distance relative to the plant gutter. Intel Realsense D415 IR- based stereo vision camera [12] was mounted in an enclosure box and installed at the top of the camera pole in a way that the angle of the camera can be changed. Camera was connected to a laptop using USB type-3 cable and Realsence Viewer software was used. The actual image of the Imaging platform is visualized in Fig. 6.

### 4.2 Data collection

RGBD camera was installed with latest firmware version and Realsense Viewer was updated to compatible software version. Then camera was reset to the manufacturer's recommended factory calibration for new cameras. Camera was used with IR emitter with power of 300, gain of 16, enabled auto exposure, and depth scale of 0.001. The full configuration of parameters is shown in Table 3.

**Table 3:** Parameters of camera configuration.

Configurations of RGB Module		Configurations of Depth Module	
Parameter	Sample value	Parameter	value
Resolution	1280×720	Resolution	1280×720
Frame Rate (FPS)	15	Frame Rate (FPS)	15
Color	RGB8	Depth	Z16
Enable Auto Exposure	1	Visual Preset	1
Exposure	156	Emitter Enabled	1
Gain	64	Enable Auto Exposure	1
Power Line Frequency	3	Exposure	33,000
Auto Exposure priority	0	Gain	16
Global Time Enabled	1	Laser Power	300
Backlight Compensation	0	Asic Temperature	44
Brightness	0	Error Polling Enabled	1
Contrast	50	Project Temperature	44
Gamma	300	Output Trigger Enabled	0
Hue	0	Depth Units	0.001
Saturation	64	Stereo Baseline	55.167847
Sharpness	50	Inter Cam Sync Mode	0
Auto White Balance	1	Global Time Enabled	1
White Balance	4600	HDR Enabled	0
		Sequence Name/Size/Id	0/2/0
		Auto Exposure Limit	200,000
		Auto Gain Limit	248
		Auto Exposure Limit Toggle	0
		Auto Gain Limit Toggle	0
		Enable Auto White Balance	0

Data were collected weekly for the right side of the third gutter in three rooms (diffused light, Smart Glass, and LLEAF). The plants in the LLEAF room were growing faster and reached maximum height at 17th week up to which they can still be imaged with our camera setup, while plants in the other two rooms could be imaged up to 21 weeks, when they reached maximum height. The platform was manually moved from the beginning of a gutter to the end with an average speed of 0.33 ms^−1^ to record the RGBD stream. For each data collection of a gutter, perpendicularity of the camera pole was ensured; then, the location and angle of the camera were set to allow the visibility of the full plant height. As the camera pole was in the middle of the trolley, full height of first few and last few plants were not recorded properly. The heights of the plants were collected manually by measuring the stem length for the right side branch of the selected plants. A mark was placed at the level of the plant top on the vertically supported wire each week, and then it was used as the base to measure the increased height the following week. Manually measured height and camera angles were recorded in .csv files, and RGBD streams were recorded as Rosbag (.bag) files.

### 4.3 Preprocessing of image data

#### 4.3.1 Image extraction

The python wrap Pyrealsense of the Realsense library [13] from Realsense@™ was used with python to process saved rosbag files. We manually selected the starting and ending frame of the stream to crop the stream to align with the starting and ending plant. First, a bag file was loaded with Realsense viewer and played with RGB image metadata. The frame numbers of the starting frame and ending frame that needed to be extracted were identified. The frame number of the very first frame was also taken, and the IDs of the first and last frame were calculated by subtracting the first frame number from first and last frame numbers that we identified (Equation *1*). Then an offset was calculated to extract the number of frames required as in Equation *2*. We used a few post-processing filters: decimation filter, temporal filter, and spatial filter from Pyrealsense library. The full python code for image frame extraction can be found in the extract_frame.py file at our GitHub repository [2]. It supports saving RGB and depth images with the same file name of stream file under directories of “depth” and “color”.

frame_id=selected_frame_number−first_frame_number

Equation 1: Calculation of frame-Id for a selected frame

$$offset=\frac{end\_frame\_id-srat\_frame\_id}{number\_of\_frames}$$

Equation 2: Calculation of offset to extract a number of frames.

**Fig. 7:**
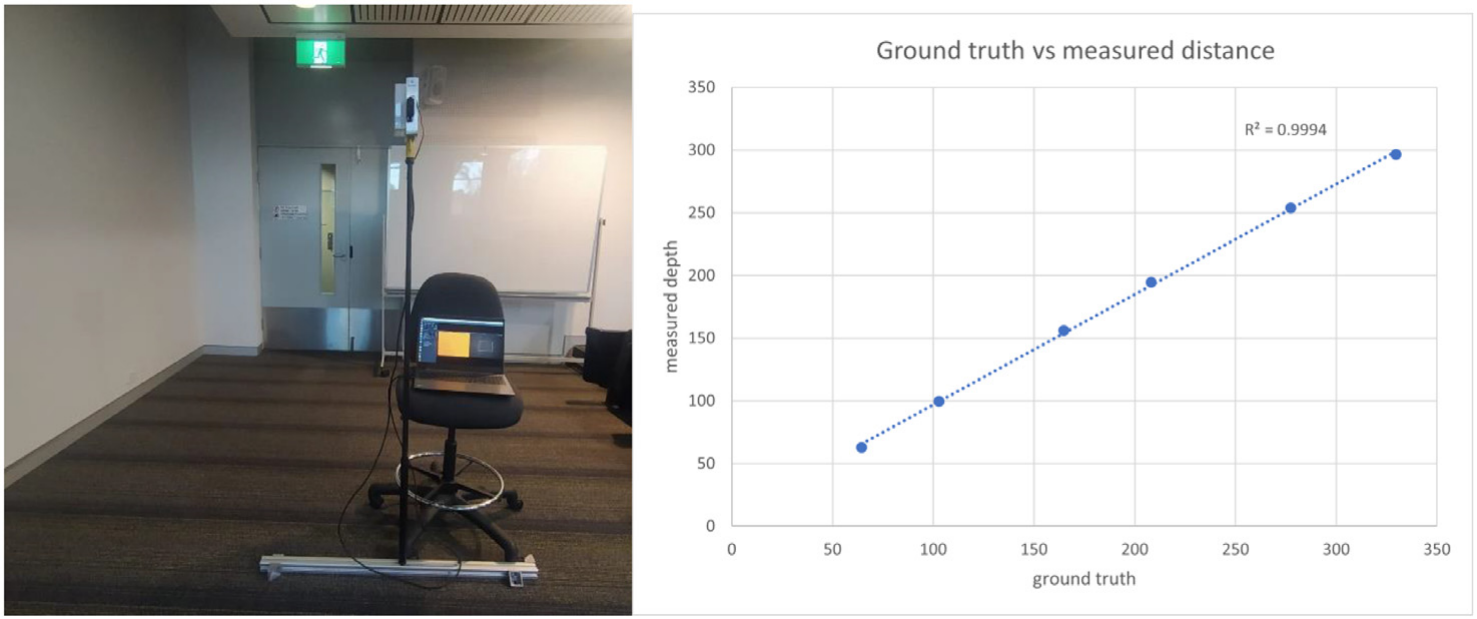
Collecting data for depth calibration error correction. Reused from Fig. 4 of [3], with permission.

As we mentioned in the data description, a calibration error of the depth camera was observed after data collection. For correcting the error, an experiment was conducted indoors. The same camera with the same configurations was used in a room facing a wall; actual and image-based distances were measured for 0.5 m - 3.0 m range and for each half a meter. Realsense depth quality tool was used for measuring the image-based depth and 80 % of the region of interest, 100 % of depth fill rate, and the camera angle closer to 0° were maintained. Then a linear regression model was fitted with the observed image-based and ground truth distance data to correct the depth calibration error of the extracted depth images. Experiment setup for data collection of depth error correction and the fitted regression model are shown in Fig. 7 while the collected data for depth error correction are shown in Table 4. The construct_3d python file in the provided GitHub project consist of application of the correction and supports visualizing images before and after depth correction. We used open3D [14] Python library for 3D reconstruction of scenes using extracted RGB and depth images and the code for both reconstruction and visualization is in the same construct_3d python file. Open3D also supports various processing techniques for 3D point clouds and can be easily integrated with the provided code for further processing the 3D scene. Examples of original RGB image, depth image, corrected depth and 3D scene are visualized in Fig. 2.

**Table 4:** Collected data for depth error correction using regression model.

Depth (cm)	Ground truth (cm)	Plane fit RMS error	Camera angle
64.438	62.8	0.37	1.42
102.774	99.5	0.5	3.04
164.697	156	0.49	0.45
208.097	194.5	0.8	3.72
277.413	253.8	0.87	1.36
329.621	296.5	0.78	3.3

## Limitations

The depth images are captured with a calibration error, and they show an overestimation of depth; however, regression-based error correction is provided to overcome this limitation.

Since the top angled view is used to focus on the full height and the top area of the plants, the middle and lower areas are not fully visible and not good for feature extraction in those areas.

The noise of the depth images was proportional to the camera to object distance, hence lower canopy level is noisier.

The imaging platform is moved manually and the speed may not be constant.

Images are captured under natural light, hence, noises such as bright spots and shading are present for some streams.

The ground truth is available only for plant height and there are missing values for three weeks.

For the data under the LLEAF film, the last four time points are not available due to the limitations of the hardware platform.

## Ethics Statement

We hereby confirm that we, the authors, have thoroughly read and adhered to the ethical requirements for publication in Data in Brief. Furthermore, we affirm that the current work does not involve human subjects, animal experiments, or any data collected from social media platforms.

## Credit Author Statement

**Namal Jayasuriya:** Conducted research design experiments, software developments and prepared the manuscript. **Oula Ghannoum**: Equally contributed in research design and manuscript preparation. **Wen Hu**: Equally contributed in research design and manuscript preparation. **Norbert Klause**: Contributed in crop maintaining and collection of manual height data. **Weiguang Liang**: Contributed in maintaining the crop, managing the crop and research activities. **Yi Guo**: Equally contributed in research design and manuscript preparation.

## Declaration of Competing Interest

The authors declare that they have no known competing financial interests or personal relationships that could have appeared to influence the work reported in this paper.

## Data Availability

PC4C_CAPSI: Image Data of Capsicum plant growth in Protected Horticulture (Original data) (ResearchDirect).
